# Isolation and Characterization of Novel Chlorella Vulgaris Mutants With Low Chlorophyll and Improved Protein Contents for Food Applications

**DOI:** 10.3389/fbioe.2020.00469

**Published:** 2020-05-19

**Authors:** Lisa Schüler, Etiele Greque de Morais, Mafalda Trovão, Adriana Machado, Bernardo Carvalho, Mariana Carneiro, Inês Maia, Maria Soares, Paulo Duarte, Ana Barros, Hugo Pereira, Joana Silva, João Varela

**Affiliations:** ^1^Marine Biotechnology Group, Centre of Marine Sciences, University of Algarve, Faro, Portugal; ^2^Allmicroalgae Natural Products S.A., Pataias, Portugal; ^3^LEPABE – Laboratory for Process Engineering, Environment, Biotechnology and Energy, Faculty of Engineering of the University of Porto, Porto, Portugal

**Keywords:** heterotrophic cultivation, microalgae, nutritional applications, pigments, protein, random mutagenesis, scale-up

## Abstract

Microalgae are widely used as food supplements due to their high protein content, essential fatty acids and amino acids as well as carotenoids. The addition of microalgal biomass to food products (e.g., baked confectioneries) is a common strategy to attract novel consumers. However, organoleptic factors such as color, taste and smell can be decisive for the acceptability of foods supplemented with microalgae. The aim of this work was to develop chlorophyll-deficient mutants of *Chlorella vulgaris* by chemically induced random mutagenesis to obtain biomass with different pigmentations for nutritional applications. Using this strategy, two *C. vulgaris* mutants with yellow (MT01) and white (MT02) color were successfully isolated, scaled up and characterized. The changes in color of MT01 and MT02 mutant strains were due to an 80 and 99% decrease in their chlorophyll contents, respectively, as compared to the original wild type (WT) strain. Under heterotrophic growth, MT01 showed a growth performance similar to that of the WT, reaching a concentration of 5.84 and 6.06 g L^−1^, respectively, whereas MT02 displayed slightly lower growth (4.59 g L^−1^). When grown under a light intensity of 100 μmol m^−2^ s^−1^, the pigment content in MT01 increased without compromising growth, while MT02 was not able to grow under this light intensity, a strong indication that it became light-sensitive. The yellow color of MT01 in the dark was mainly due to the presence of the xanthophyll lutein. On the other hand, phytoene was the only carotenoid detected in MT02, which is known to be colorless. Concomitantly, MT02 contained the highest protein content, reaching 48.7% of DW, a 60% increase as compared to the WT. MT01 exhibited a 30% increase when compared to that of the WT, reaching a protein content of 39.5% of DW. Taken together, the results strongly suggest that the partial abrogation of pigment biosynthesis is a factor that might promote higher protein contents in this species. Moreover, because of their higher protein and lower chlorophyll contents, the MT01 and MT02 strains are likely candidates to be feedstocks for the development of novel, innovative food supplements and foods.

## Introduction

The consumer demand for health-promoting and nutritional-rich foods has been increasing over the last few years. Microalgae are a sustainable biological resource with a well-balanced biochemical profile, rich in protein and bioactive compounds such as carotenoids and essential fatty acids that provide potential benefits for human health (Lucas et al., 2018). Nevertheless, from the thousands of microalgal strains currently described and identified, only a narrow number of strains are currently approved for human consumption. In the EU, *Arthrospira platensis* (“spirulina”) and *Chlorella vulgaris* are approved for human consumption due to a long history of safe use, being well-established in the market, while odontella aurita and tetraselmis chui were recently approved as novel foods by the european food safety authority (EU, 2017/2470).

Microalgal biomass is widely commercialized worldwide in the nutraceutical sector as food supplements (e.g., tablets and capsules), while in the food market they are normally incorporated as a natural food colorant or as a healthy supplement, able to enhance the nutritional value of conventional food products (e.g., bars, pasta and cookies; Sahni et al., 2019). Nevertheless, the incorporation of microalgae in food products faces challenges mainly due to their organoleptic characteristics, including a strong color, taste and odor (Lafarga, 2019). The sensory attributes of foods are directly linked to the consumer acceptance whereby the color is the first parameter observed by the consumer and can be decisive for whether or not to include the food in their diet (Delwiche, 2012). Therefore, microalgal-based food products that are usually green in color comes with very low sensorial acceptance by the consumer. Moreover, chlorophyll, the pigment responsible for the green color of microalgae and higher plants, usually imparts a grassy taste to tea (van Lelyveld and Smith, 1989). Therefore, these less favorable organoleptic characteristics of microalgal biomass need to be modified in order to improve its acceptance in food products.

Alternative strategies to improve the organoleptic qualities of food containing microalgal biomass have included the extraction of the target compounds with the concomitant removal of chlorophyll or the addition of ingredients such as chocolate to improve the final flavor and color (Lucas et al., 2018). Another option could be isolation of novel microalgal strains with improved organoleptic characteristics. Random mutagenesis is an interesting cell modification tool for food applications, as it is not considered a method that generates genetic modified organisms (GMOs), because it does not introduce any foreign genetic material into the target cell (Zimny et al., 2019, directive 2001/18/ec). By exposure of the target cells to physical (e.g., UV light) or chemical mutagenic agents (e.g., ethyl methane sulfonate), strains with improved characteristics are generated. Upon mutagenesis, it is important to apply a selection procedure to screen for the desired mutants, e.g., abiotic stress factors such as light intensity. Furthermore, when the genes of the carotenoid biosynthetic pathway are targeted, specific inhibitors can be used such as compactin, diphenylamine, nicotine or norflurazon (Cordero et al., 2011; Chen et al., 2017; Huang et al., 2018).

Accordingly, the aim of this work was to develop chlorophyll-deficient mutants of *C. vulgaris* by chemically induced random mutagenesis in order to obtain biomass with different pigmentations for nutritional applications. The heterotrophic growth performance under light and dark conditions of wild type (WT) and established mutants was evaluated as well as their proximate biochemical composition and pigment profile. One of the mutants was scaled up to evaluate the growth performance in 5-L and 200-L fermenters and determine their feasibility as future feedstocks for the food industry.

## Materials and Methods

### Wild Type Inoculum and Growth

*Chlorella vulgaris* was obtained from Allmicroalgae Natural Products S.A. culture collection. The cryopreserved cultures stored in liquid nitrogen were transported to the Center of Marine Sciences (University of Algarve) on dry ice. The inoculum was transferred to a 50 mL centrifuge tube containing 20 mL of culture medium, comprising 0.1% glucose, 0.25% yeast extract and 0.5% peptone. The culture was later divided into several 250-mL Erlenmeyer flasks with a working volume of 50 mL containing the same medium and incubated in an orbital shaker at 28 ± 2°C under constant shaking (100 rpm).

### Random Mutagenesis and Selection of Chlorophyll-Deficient Mutants

Cells of *C. vulgaris* growing exponentially (3.2 × 10^6^ cells mL^−1^) were concentrated 10-fold by centrifugation (3,000 *g*, 3 min) and treated with 150, 200, 250, 300, 350, and 400 mM ethyl methane sulfonate (EMS, Merck, USA) for 1 h under mild agitation in the dark (FAO/IAEA, 2018). By addition of sodium thiosulfate to a final concentration of 5%, the reaction of EMS was stopped, and cells were pelleted by centrifugation at 3,000 *g* for 3 min. Cells were washed thrice with sterile distilled water and incubated for 24 h in the dark to prevent light-dependent DNA repair. For the determination of the cell survival rate, cells were plated onto Plate Count Agar (PCA; VWR, Portugal) in serial dilutions and incubated at 30°C for 72 h in the dark. The mutant selection was carried out by visual observation of the plates in dim light. A colony with yellow color was picked, sub-cultured several times on PCA plates and subsequently transferred into liquid media. This yellow mutant was grown to exponential phase and subjected to a second round of random mutagenesis using 300 mM EMS. This time, mutant selection was performed on PCA plates with the carotenoid biosynthesis inhibitor norflurazon, which blocks phytoene desaturase (Breitenbach et al., 2001; Koschmieder et al., 2017). To choose the lowest concentration that inhibits cell growth of the mutant, cells were previously spread onto 2, 4, 8, and 10 μM of norflurazon plates. Only at 10 μM the authors obtained plates without any colonies, whereas lower concentrations led to a lawn of cells. Therefore, upon mutagenesis, cultures were plated onto PCA plates containing 10 μM of norflurazon and incubated at 30°C in the dark for 1 week. Herbicide-resistant white colonies were sub-cultured several times, first on plates containing norflurazon and afterwards on plates without herbicide to confirm the phenotypic stability of the mutants.

### Experimental Trials in Erlenmeyer Flasks

Experimental trials were conducted to evaluate the heterotrophic growth performance and biochemical composition of WT and established mutants under dark and light conditions. The trial was conducted in 250-mL Erlenmeyer flasks, with a final working volume of 50 mL, using a heterotrophic basal medium (HM; Barros et al., 2019) supplemented with glucose (20 g L^−1^). Cultures were then placed in two orbital shakers at 30°C and 200 rpm. A spotlight was kept on top of one orbital shaker using a photon flux density of 100 μmol m^−2^ s^−1^ (light condition), while the other orbital shaker was covered with aluminum foil (dark condition). All experimental trials were carried out in triplicate.

### Growth Comparison of Wild Type vs. Mutant in 5-L and 200-L Fermenters

The seed for heterotrophic growth was obtained sequentially in 50- and 250-mL cultures in, respectively, 250-ml and 1000-mL Erlenmeyer flasks, in order to reach a volume of 5 L in a bench-top fermenter (New Brunswick BioFlo® CelliGen®115; Eppendorf AG, Hamburg, Germany), which was later used to inoculate a 200-L fermenter, developed and assembled in-house. Temperature in both fermenters was maintained at 30°C and pH at 6.5 by addition of ammonia solution (24% m m^−1^). As in the Erlenmeyer flask tests, HM medium was used (Barros et al., 2019), but glucose was added in fed batch so that a non-limiting concentration of 1 to 20 g L^−1^ was kept. Samples were collected aseptically for supernatant analysis or biomass concentration analysis. Throughout the growing period the air inlet flowrate was adjusted to maintain ~1 vvm. Accordingly, the agitation rate ranged from 100 to 1,200 rpm, so that the dissolved oxygen in the medium was not a limiting factor for culture growth.

### Sampling and Growth Assessment

Sampling of each culture was done twice a day in order to analyze growth parameters, namely optical density (OD) at 600 nm using Genesys 10S UV-Vis spectrophotometer (Thermo Fisher Scientific, Massachusetts, USA), pH and optical microscopy (Zeiss Axio Scope A1, Oberkochen, Germany).

Dry weight (DW) determination was only possible for the samples of cultures grown in fermenters. Culture samples were filtered using pre-weighed 0.7 μm GF/C 698 filters (VWR, Pennsylvania, USA) and dried at 120°C until constant mass was obtained using a DBS 60–30 electronic moisture analyzer (KERN & SOHN GmbH, Balingen, Germany). All dry weight samples were washed with demineralized water to remove growth medium salts. Whenever the previous procedure could not be carried out, a DW vs. optical density correlation developed in-house for this strain was used. Biomass productivity was obtained by equation 1 and growth rate by equation 2.

(1) $$\begin{array}{r} P\;\left(g\;L^{-1}\;d^{-1}\right)\;=\;\frac{DW_{f}-DW_{i}}{t_{f}-t_{i}} \end{array}$$

(2) $$\begin{array}{r} \mu \;\left(d^{-1}\right)\;=\;\frac{ln\left(DW_{f}/DW_{i}\right)}{t_{f}-t_{i}} \end{array}$$

### Proximate Composition

The protein content was determined by CHN elemental analysis, according to the procedure provided by the manufacturer using a Vario el III (Vario EL, Elemental Analyzer system, GmbH, Hanau, Germany). The final protein content was calculated by multiplying the percentage of nitrogen by 6.25.

The lipid content was determined using the Bligh and Dyer (1959) method described in Pereira et al. (2011) with minor modifications. Briefly, freeze dried samples were extracted with methanol through bead-milling with glass beads, using a Retsch MM 400 mixer mill at 30 Hz for 3 min to ensure effective cell disruption. The tubes were centrifuged (10,000 *g*) and the supernatants were collected to new vials. The pellets suffered a second extraction and both methanol supernatants were pooled. Chloroform and water were added to the methanol (2:1:2) and the tubes were vortexed for 5 min. Afterwards, the samples were centrifuged to obtain a biphasic system and the lipid extract was separated. A known volume of the extracts was transferred to pre-weighed tubes, evaporated and weighted in order to determine the lipids gravimetrically.

The ash content was determined by burning the freeze-dried biomass in a furnace (J. P. Selecta, Sel horn R9-L, Barcelona, Spain) at 550°C for 6 h. The carbohydrate content was determined by difference of the remaining macronutrients.

### Chlorophyll Content

A culture volume corresponding to 10 mg of biomass was taken from each sample and centrifuged for 15 min, at 2,547 *g* (HERMLE Labortechnik GmbH, Wehingen, Germany). Chlorophyll extraction was performed in acetone by successive zirconia bead milling. The supernatant was collected by centrifugation and re-extraction of the biomass was performed until colorless. The absorbance of the supernatant was measured in a Genesys 10S UV-Vis spectrophotometer (Thermo Scientific, Massachusetts, USA) at 630, 647, 664 and 691 nm. The chlorophyll *a* content was then estimated according to the following equation by Ritchie (2008):

(3) $$\begin{array}{r} {Chl}_{a}=-0.3319\;Abs_{630}\;-\;1.7485\;Abs_{647}\;+\;11.9442\;Abs_{664}\\ \;-\;1.4306\;Abs_{691} \end{array}$$

### Carotenoid Profile

The extraction of carotenoids was carried out on ice and under dim light to avoid oxidation. Approximately 5 mg of freeze-dried biomass was weighed in a glass vial, ~0.6 g of glass beads (425–600 μm) and 1 mL of ice-cold methanol containing 0.03% butylhydroxytoluene (BHT) were added. Cells were disrupted using a Retsch MM 400 mixer mill at 30 Hz for 3 min. To collect the supernatant, the samples were centrifuged for 3 min at 21,000 *g*. The remaining biomass was extracted repeatedly with 1 mL of methanol/BHT by vortexing for 10 s, followed by centrifugation until both the pellet and the supernatant became colorless. The extracts were combined, and methanol was evaporated under a gentle nitrogen flow. Prior to HPLC analysis, the extracts were resuspended in 1 mL of methanol and filtrated through 0.22 μm PTFE filter to remove suspended particles.

Carotenoid analysis was performed by HPLC as described previously (Schüler et al., 2020). Briefly, a Dionex 580 HPLC System (DIONEX Corporation, USA) consisting of a PDA 100 Photodiode-array detector, STH 585 column oven set to 20°C and a LiChroCART RP-18 (5 μm, 250 × 4 mm, LiChrospher) column was used. Carotenoid separation was achieved using a mobile phase composed of solvent A acetonitrile:water (9:1; v v^−1^) and solvent B ethyl acetate with the following gradient: 0–16 min, 0–60% B; 16–30 min, 60% B; 30–32 min 100% B and 32–35 min 100% A. All carotenoids were detected at 450 nm and 280 nm and analyzed with Chromeleon Chromatography Data System software (Version 6.3, ThermoFisher Scientific, Massachusetts, USA). The quantification was carried out using calibration curves of neoxanthin, violaxanthin, lutein, zeaxanthin and β-carotene standards (Sigma-Aldrich, Portugal). Phytoene was identified by its specific absorbance profile at 280 nm and only quantified as equivalent to lutein. Injection volume of both extracts and standards was 100 μL.

### Statistical Analysis

Statistical analyses were performed with R software (version 3.6.1). Statistical significance was tested using analysis of variance (one-way ANOVA) and Tukey HSD *post-hoc* at a 0.05 probability level.

## Results and Discussion

### Development of Mutants

**Figure 1:**
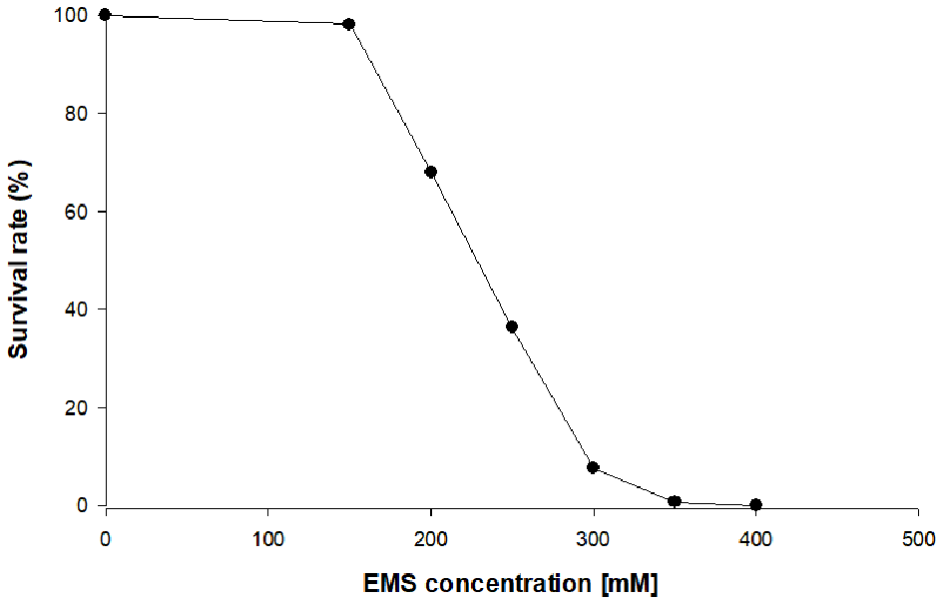
Survival rate of heterotrophic *Chlorella vulgaris* upon exposure to different ethyl methane sulfonate (EMS) concentrations on plate count agar (PCA) plates.

In the first stage of this work, chlorophyll-deficient mutants of *C. vulgaris* were obtained by random mutagenesis using the alkylating agent ethyl methane sulfonate (EMS). Different concentrations of EMS were tested on the WT to find the concentration, which resulted into a survival rate between 5 and 10% (Figure 1). The selection of the correct survival rate is critical to increase the likelihood that the survivors contain at least one mutation, but also to avoid the selection of cells containing multiple mutations, which are often detrimental to growth. The selection and further scale up of the mutants were carried out in the absence of light and with glucose as carbon source to suppress the need for energy supply via photosynthesis, and thus promoting the growth of chlorophyll-deficient mutants. After treatment of the cells with a concentration of 300 mM of EMS, a yellow colony indicating the absence of chlorophyll emerged onto the plate. The repeated sub-cultivation on solid media of this mutant, MT01, confirmed the stability of the yellow color throughout 10 generations. Most probably, a mutation in the photosynthetic machinery is the reason for the reduction of chlorophyll in this mutant (Tiwari et al., 2019).

Thereafter, a second random mutagenesis was conducted on MT01, with subsequent selection of mutants by their resistance to the carotenogenic pathway inhibitor norflurazon. A wide range of concentrations of norflurazon was tested to find out that 10 μM was the minimal lethal concentration to MT01. This selection procedure gave rise to white colonies with resistance to the bleaching herbicide norflurazon. After sub-cultivation, only one mutant maintained the white color when the herbicide was removed from the media over 10 generations. This mutant, MT02, most probably contains an irreversible mutation in the phytoene desaturase gene leading to the inhibition of the following steps within the carotenoid and/or plastoquinone biosynthetic pathways (McCarthy et al., 2004; Qin et al., 2007). Other studies on *Chlorella zofingiensis* and *Chlorella sorokiniana* used a similar approach to obtain mutants with accumulation of zeaxanthin or lutein, respectively (Chen et al., 2017; Huang et al., 2018). In those cases, the inhibitors diphenylamine or nicotine were used to select for mutations in genes coding for enzymes involved in carotenoid biosynthesis.

### Wild Type vs. Mutants in Dark and Light Conditions

#### Growth Performance

**Figure 2:**
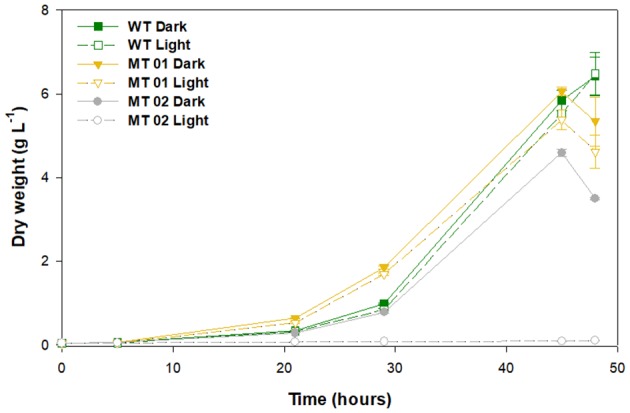
Growth curves of wild type and mutants, under light and dark conditions grown in 250-mL Erlenmeyer flasks for 48 h.

*C. vulgaris* WT and mutants were grown in 250-mL Erlenmeyer flasks under light and dark conditions, to assess the effect of light on growth performance and biomass color (Figure 2). After a lag phase of about 20 h the cultures grew exponentially until the depletion of glucose, which led to cell death after 48 h.

The WT along with the yellow mutant MT01, both in the dark, reached the highest DW after 45 h of growth, 5.84 and 6.06 g L^−1^, respectively. Under light conditions, the WT and MT01 achieved a similar DW (*p* > 0.05), 5.52 and 5.38 g L^−1^, respectively, but significantly lower than that obtained under dark conditions (Figure 2). Several pale-green *C. vulgaris* mutants reported in literature also showed biomass productivity similar to that of the WT strain used, however, under autotrophic conditions (Shin et al., 2016; Dall'Osto et al., 2019). Furthermore, those mutants showed with increasing light intensity higher biomass productivities (up to a 68% increase) than that of the WT. Those studies further showed that the changes observed not only improved growth performance, but also the pigment profile, at the cost of higher sensitivity to light. Interestingly, all these phenotypes were associated to smaller antenna sizes in the photosynthetic machinery of the mutants (Shin et al., 2016; Dall'Osto et al., 2019).

The white mutant MT02 displayed a significantly lower biomass concentration in the dark compared to the WT and MT01 (*p* < 0.05), attaining a maximum DW of 4.59 g L^−1^ after 45 h of growth. Moreover, MT02 was not able to grow under light conditions, achieving only 0.08 g L^−1^ of DW at the end of the assay. Similarly, Kamiya (1985) also described light, particularly blue light, as inhibitory for growth, cell division and glucose uptake for colorless *Chlorella* mutants. Nonetheless, in the dark, MT02 displayed a promising growth performance, which was statistically indistinguishable from that of the WT (*p* > 0.05). In spite of enhancing pigment content, exposure to excess light might lead to a more or less noticeable growth inhibition, which in this case was observed not only in the white MT02 mutant growth, but also in the WT and yellow MT01 mutant cultures exposed continuously to light.

#### Pigment Profile

**Figure 3:**
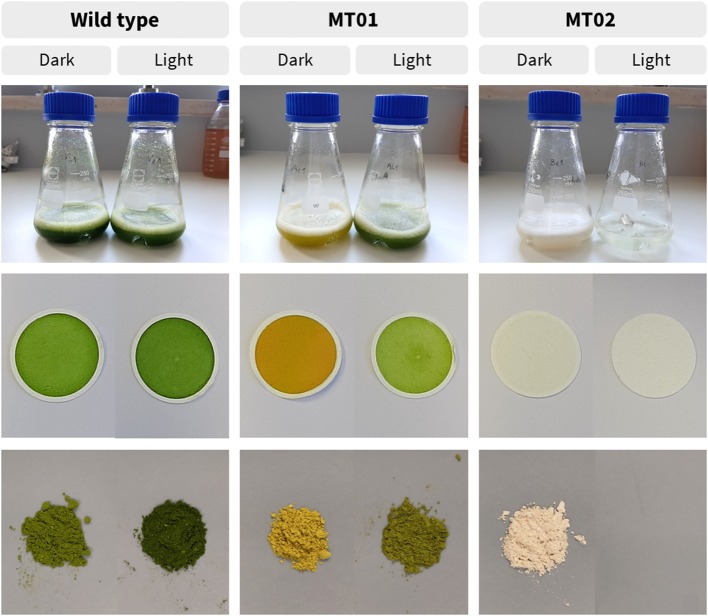
Different coloration of wild type and mutant cultures, dry weight filters and freeze-dried biomass, grown under light and dark conditions in 250-mL Erlenmeyer flasks, after 42 h.

Macroscopically, WT cultures displayed a green color and acquired a more intense green color when grown under a spotlight (Figure 3). MT01 cultures presented an intense yellow color under dark conditions, which was reversed back to green when cultures were exposed to light conditions. On the other hand, MT02 cultures exhibited a white tonality and absence of any other color under dark conditions, while no biomass was produced under light conditions.

**Figure 4:**
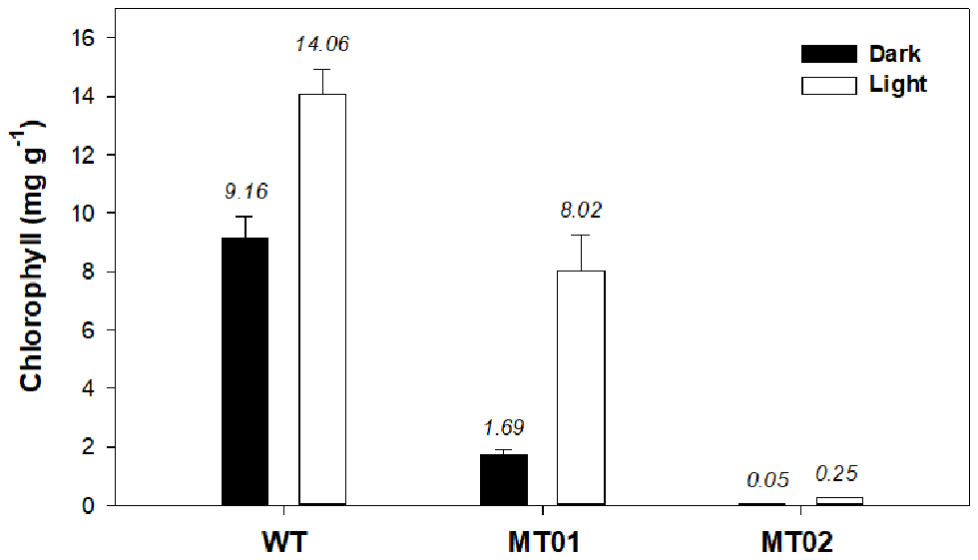
Chlorophyll content (mg g^−1^) of *Chlorella vulgaris* wild type and mutants grown in 250-mL Erlenmeyer flasks under dark and light conditions. Values are given as means ± standard deviation of three biological replicates (*n* = 3).

In order to characterize the color of WT and mutant strains under light and dark conditions, the chlorophyll and carotenoid content of the cultures were analyzed. It is evident that light significantly increased the chlorophyll content of WT and mutant cultures (*p* < 0.05) (Figure 4). Although, MT01 and WT exhibited equivalent growth performances (*p* > 0.05), MT01 contained significantly lower chlorophyll content than the WT (*p* < 0.05) under both light and dark conditions. WT cultures displayed the highest chlorophyll content, 9.16 mg g^−1^ under dark conditions, which was enhanced to 14.06 mg g^−1^ in the presence of light. MT01 cultures grown in the dark registered 1.69 mg g^−1^ of chlorophyll, while under light exposure 8.02 mg g^−1^ of chlorophyll was detected, which granted them the green coloration. In fact, no significant differences were observed between the chlorophyll content of WT grown in the dark and the light grown MT01 displaying a pale green color (*p*>0.05). This is in agreement with studies of *C. vulgaris*, where EMS-induced light green mutants with a 50% reduced chlorophyll content compared to the WT were selected (Shin et al., 2016; Dall'Osto et al., 2019). However, cultures in those studies were grown under autotrophic conditions as the objective was to enhance biomass productivities and photosynthetic efficiencies.

The MT02 mutant, however, displayed only residual chlorophyll contents grown in the dark (0.045 mg g^−1^). Although not easily visible, after some days of light exposure, MT02 started to acquire a pale green tonality, which was evidenced by the detection of an increased chlorophyll content in the biomass as compared with the dark cultured biomass (0.25 mg g^−1^; *p* < 0.05). This is in accordance with studies on EMS-induced white mutants of *Chlamydomonas reinhardtii* and *Chlorella vulgaris*, which showed a pale green color due to a 40-fold decrease in chlorophyll content compared to the WT (Kamiya, 1985; McCarthy et al., 2004). However, with the mutants developed in this work, which are heterotrophically cultivated, it is possible to maintain a stable non-green color under dark conditions.

The carotenoid profile of *C. vulgaris* WT was mainly composed of lutein and β-carotene, while neoxanthin, violaxanthin and zeaxanthin were only detected in minor quantities (Table 1). The carotenoid profile of MT01 showed the same characteristics as compared with the WT, however, with lower contents of 0.93 ± 0.01 and 1.70 ± 0.13 mg g^−1^ DW in the dark, respectively. As lutein is the major carotenoid, this can explain the yellow color of MT01 under dark conditions (Figure 3). Huang et al. (2018) also obtained a yellow *Chlorella* mutant by random mutagenesis with similar growth performances of the wild type strain. That mutant strain displayed a dysfunction in carotenoid ketolase enzyme, which prompted zeaxanthin accumulation (up to 7.00 mg g^−1^) enhanced by high-light irradiation, nitrogen depletion and glucose feeding. Those treatments also led to the accumulation of other carotenoids, such as β-carotene (7.18 mg g^−1^) and lutein (13.81 mg g^−1^), which together imparted their yellowish hues to the biomass. In addition, Dresbach and Kowallik (1974), which also established a chlorophyll-free *C. vulgaris* mutant pointed out that carotenoid biosynthesis might be enhanced by permanent irradiation with blue light. Moreover, several positive effects on human health such as the reduced risk for cardiovascular disease and age-related macular degeneration as well as cancer prevention have been attributed to lutein (Astorg, 1997; Ma et al., 2012; Han et al., 2015). Therefore, it could be interesting to study the accumulation of this pigment in MT01 by testing other stressing or stimulating factors, such as nitrogen depletion, glucose feeding and other light wavelengths or intensities.

**Table 1:** Carotenoid content of *Chlorella vulgaris* WT and chlorophyll-deficient mutants MT01 and MT02 grown in 250 mL Erlenmeyer flasks under light and dark conditions.

**Culture**	**Condition**	**Neoxanthin**	**Violaxanthin**	**Lutein**	**Zeaxanthin**	**β-carotene**	**Phytoene**
		**(mg g^**−1**^ DW)**	**(mg g^**−1**^ DW)**	**(mg g^**−1**^ DW)**	**(mg g^**−1**^ DW)**	**(mg g^**−1**^ DW)**	(mg g^**−1**^ DW)^*^
WT	Dark	0.085 ± 0.008^b^	0.043 ± 0.007^a^	1.280 ± 0.077^b^	0.007 ± 0.001^b^	0.284 ± 0.036^b^	0.194 ± 0.010^e^
	Light	0.181 ± 0.012^a^	0.033 ± 0.007^ab^	1.853 ± 0.060^a^	0.010 ± 0.001^a^	0.585 ± 0.047^a^	0.252 ± 0.012^d^
MT01	Dark	0.005 ± 0.001^d^	0.033 ± 0.010^ab^	0.858 ± 0.003^c^	0.003 ± 0.001^c^	0.034 ± 0.001^c^	0.320 ± 0.004^c^
	Light	0.038 ± 0.009^c^	0.016 ± 0.006^b^	1.167 ± 0.079^b^	0.009 ± 0.001^ab^	0.322 ± 0.026^b^	0.363 ± 0.008^b^
MT02	Dark	0	0	0	0	0	0.414 ± 0.010^a^
	Light	n.a.	n.a.	n.a.	n.a.	n.a.	n.a.

*Different letters indicate significant differences (p > 0.05) between strains and treatments. Values are given as means ± standard deviation of three biological replicates (n = 3)*.

*n.a., not analyzed due to insufficient biomass sample*.

* *Values calculated as lutein-equivalent contents*.

Increased light intensity seems to promote the induction of carotenoids in both WT and MT01 by about 1.6-fold (Table 1). This is most probably related with the function of carotenoids, as they are important pigments involved not only in light harvesting, but also in the protection of the photosynthetic apparatus from excess light (Mulders et al., 2014). As expected, the content of violaxanthin decreased with the concomitant increase of the photoprotective xanthophyll zeaxanthin (Table 1). Remarkably, the content of β-carotene in MT01 cultivated under light conditions increased 10-fold compared with cells under dark conditions, confirming the importance of this carotenoid as photoprotective pigment in this microalga. Conversely, as its white color indicated already, all colored carotenoids were absent in the MT02 mutant; the only carotenoid detected was the colorless phytoene with 2-fold higher concentrations as compared with the WT under dark conditions (Table 1). Phytoene is a linear carotenoid without a conjugated system of double bonds, which has already been reported to be ineffective in photoprotection (León et al., 2005). This is most probably the reason why MT02 was not able to grow under light conditions. Phytoene, however, has gained interest in the cosmetic industries due to its absorption of UV radiation, anti-inflammatory and anti-oxidant effects (Meléndez-Martínez et al., 2018). Therefore, it would be interesting to study the accumulation of this carotenoid in the *C. vulgaris* MT02 strain under specific growth conditions to maximize its production.

#### Proximate Composition of Main Macronutrients

The comparison of the composition of main macronutrients revealed significant differences between WT, MT01 and MT02 in terms of protein, ash, and carbohydrate contents (Table 2). MT02 grown in the dark displayed the highest protein content, 48.7% of DW, followed by MT02 grown in the light and dark conditions, 45.5 and 39.5% of DW, respectively (*p* < 0.05). The WT displayed the lowest protein content under light and dark conditions, 35.3 and 30.5% of DW, respectively (*p* < 0.05). On the other hand, the highest carbohydrate content (48.8 and 42.2% of DW, in the dark and in the light, respectively) was achieved by the WT (*p* < 0.05), while MT01 and MT02 presented similar carbohydrate contents (27.1–32.0% of DW; *p* > 0.05). Interestingly, despite the great variations found in chlorophyll content between cultures and conditions, no significant differences in total lipid content were detected, which ranged from 14.3 to 18.4% of DW in all cultures and conditions (*p* > 0.05). The WT revealed the lowest ash content (5.4 and 6.6% of DW in the dark and in the light, respectively), followed by MT01 and MT02 grown in the dark (9.3–10.1% of DW), whereas MT02 grown under light conditions displayed the highest ash content (12.7% of DW; *p* < 0.05). The conditions (light vs. dark) affected protein, carbohydrate and ash significantly, resulting in higher content of both protein and ash, and lower content of carbohydrates, when cells were exposed to light (*p* < 0.05).

**Table 2:** Proximate composition of macronutrients of *Chlorella vulgaris* WT and mutants presented as percentage of dry weight.

**Culture**	**Condition**	**Proteins**	**Lipids**	**Carbohydrates**	**Ashes**
		**(% DW)**	**(% DW)**	**(% DW)**	**(% DW)**
WT	Dark	30.5 ± 0.8^e^	15.4 ± 1.9^a^	48.8 ± 2.9^a^	5.4 ± 0.5^d^
	Light	35.3 ± 0.4^d^	15.8 ± 1.5^a^	42.2 ± 1.8^b^	6.6 ± 0.7^c^
MT01	Dark	39.5 ± 0.9^c^	18.4 ± 1.8^a^	32.0 ± 1.1^c^	10.1 ± 0.2^b^
	Light	45.5 ± 0.8^b^	14.3 ± 2.3^a^	27.5 ± 3.3^c^	12.7 ± 0.4^a^
MT02	Dark	48.7 ± 1.3^a^	14.9 ± 2.4^a^	27.1 ± 2.1^c^	9.3 ± 0.2^b^
	Light	n.a.	n.a.	n.a.	n.a.

*Different letters indicate significant differences (p > 0.05) between strains and treatments. Values are given as means ± standard deviation of three biological replicates (n = 3)*.

*n.a., not analyzed due to insufficient biomass sample*.

Both higher amounts of proteins and lower amounts of chlorophyll detected in both mutants may suggest a truncated chlorophyll antenna size of the photosystems as reported in other chlorophyll-deficient mutants (Polle et al., 2002; Shin et al., 2016; Dall'Osto et al., 2019). Those chlorophyll-deficient mutants have been characterized with similar or even higher protein levels, namely chlorophyll-binding proteins and thylakoid membrane proteins (Polle et al., 2002; Gu et al., 2017). Furthermore, a previous report revealed that higher light exposure induces the accumulation of proteins; thus, in this case, low light might have induced the synthesis of larger photosynthetic units, resulting in higher protein content in the light (Seyfabadi et al., 2011). While the higher content of carbohydrate found in the dark conditions was probably due to the accumulation of polysaccharides such as starch. In addition, increased ash content in chlorophyll-free biomass has also been previously reported (Li et al., 2016), suggesting that the mineral metabolism might have also been affected in the mutants. Overall, WT cultures revealed proximate composition values within those previously reported for *C. vulgaris* grown in heterotrophic conditions (Kim et al., 2019; Canelli et al., 2020), while MT01 and MT02 displayed significantly higher protein contents. Therefore, the low ash associated with high protein contents of mutants, adds to these cultures improved nutritional profiles with commercial interest for their application as feedstocks for food products.

### Scale-Up Case Study: MT01 Growth Validation in 5-L and 200-L Fermenters

**Figure 5:**
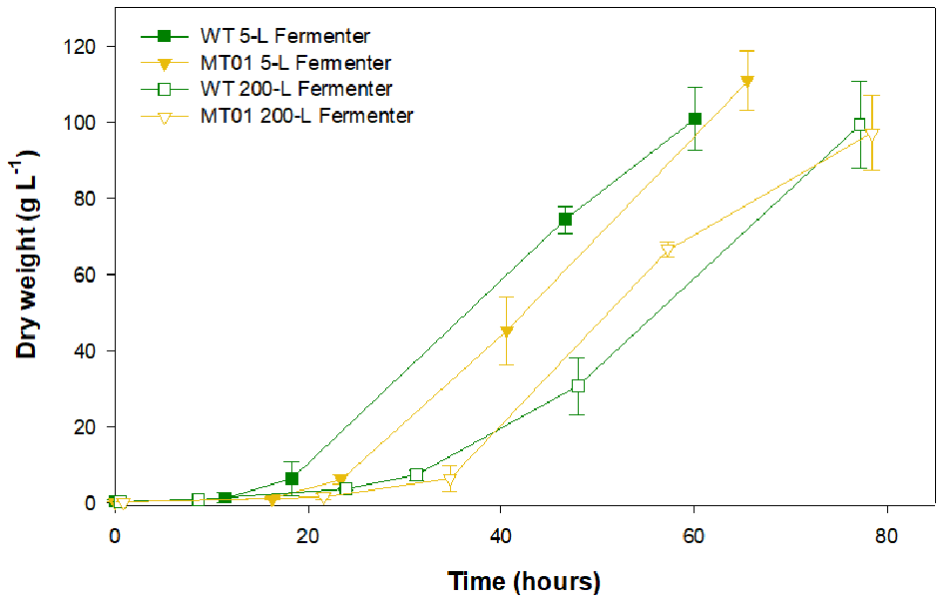
Growth curves of wild type vs. MT01 mutant in 5-L and 200-L fermenters. Values are given as means ± standard deviation of three biological replicates (*n* = 3).

In order to validate the previous results, the WT and MT01 growth performance was compared at a larger scale in 5-L and 200-L fermenters (Figure 5).

In the 5-L fermenters, growth was similar for both strains (*p* > 0.05) reaching a maximum DW of 100.94 and 110.85 g L^−1^ for the WT and MT01 cells, respectively, ~60 h upon inoculation. Similarly, no significant differences (*p* > 0.05) were observed in the growth of MT01 and WT in the 200-L fermenters as shown by the key process indicators (KPI; Table 3). Final DW here obtained was of 99.39 and 97.13 g L^−1^ for WT and MT01 strains, respectively, after ~75 h. These values are below those previously reported for the WT strain of 174.5 g L^−1^ (Barros et al., 2019). Nevertheless, the aforementioned dry weight was obtained after 7 days of growth, whereas in this run only 3 days are considered. A similar scale-up case study for a mutant of *Chlorella pyrenoidosa* was obtained by Song et al. (2018). In this case, the mutant obtained yielded 81.9 and 84.9 g L^−1^ of biomass in the 5-L and 2,000-L fermenters, respectively. As in this study, the authors point out to the homogeneity and growth patterns of their mutant upon scale-up as a strong indicator of the suitability of the mutant strain for industrial biomass production.

**Table 3:** Mean and maximum biomass productivities and growth rates of *Chlorella vulgaris* WT and mutant MT01 in 5- and 200 L fermenters.

**Strain/fermenter**	**Mean productivity**	Batch maximum productivity^*^	**Mean specific growth rate**	Batch maximum specific growth rate^*^
	**(g L^**−1**^ d^**−1**^)**	**(g L^**−1**^ d^**−1**^)**	**(d^**−1**^)**	**(d^**−1**^)**
WT 5-L Ferm	42.44 ± 5.31^a^	48.22	2.67 ± 0.32^ab^	2.92
MT01 5-L Ferm	41.03 ± 1.56^a^	42.11	2.98 ± 0.04^a^	3.01
WT 200-L Ferm	30.98 ± 2.25^b^	33.06	2.38 ± 0.08^b^	2.47
MT01 200-L Ferm	30.07 ± 1.47^b^	31.73	2.46 ± 0.26^ab^	2.64

*Same letters in superscript after the values denote significant statistical differences (p > 0.05) between values on the same column. Values are given as means ± standard deviation of three biological replicates (n = 3)*.

* *Batch maximum productivity and batch maximum specific growth rate correspond to maximum mean productivity and mean specific growth rate obtained among the three replicates, respectively*.

Concurrently, there were no statistical differences (*p* > 0.05) in the maximum nor in the average specific growth rate of WT and MT01 growth in the scales tested: 5 L and 200 L. This is an excellent indicator of the robustness of this mutant for industrial scale heterotrophic production. Maximum productivities were also similar for both strains throughout scale-up (*p* > 0.05). On the other hand, the average productivity was higher (*p* < 0.05) for both strains in the 5-L fermenter compared to the 200-L, given the shorter lag phase observed in these growth curves. In fact, the KPI for the WT and MT01 strains in the 200-L fermenter are well in accordance with the previously reported for the WT grown in the same 200-L fermenter – productivity of 27.54 ± 5.07 g L^−1^ d^−1^ and mean growth rate of 0.92 ± 0.11 d^−1^ (Barros et al., 2019). Furthermore, the biomass productivity and specific growth rate obtained for the MT01 strain were higher than those previously obtained for a *C. pyrenoidosa* mutant (19.68 g L^−1^ d^−1^ and 1.44 d^−1^, respectively) using a reactor with a volume of 2,000 L (Song et al., 2018).

## Conclusions

The established *Chlorella vulgaris* strains with yellow (MT01) and white (MT02) colors showed high biomass productivities comparable to the wild type. The color change in MT01 and MT02 cells were due to a 5- and 180-fold decrease in chlorophyll contents and the presence of lutein and phytoene, respectively, when the cells were grown heterotrophically in the dark. Both mutants displayed improved protein contents compared to that of the WT with a 60% increase under heterotrophic growth. MT01 was successfully scaled up to industrial 200-L fermenters, reaching a concentration of about 100 g DW L^−1^. Because of this growth performance as well as improved organoleptic and nutritional characteristics, both new strains MT01 and MT02 show a high potential for applications in the food and nutraceutical industries for novel products based on microalgal biomass.

## Data Availability Statement

The datasets generated for this study are available on request to the corresponding author.

## Author Contributions

LS, EG, and MT conceived, designed, and performed the experiments, prepared the figures/tables, and wrote the manuscript. AM and PD performed the experiments on the generation of the mutants and drafted the manuscript. AM and MS performed the growth trials and fermentation experiments and drafted the manuscript. MC and IM performed the analysis of the proximate biomass composition, prepared figures/tables, and drafted the manuscript. BC performed the fermentation experiments, statistical analysis, and drafted the manuscript. HP and AB conceived, designed, and supervised the experiments and wrote the manuscript. JV and JS conceived and designed the experiments, reviewed the manuscript, and contributed to the funding. All authors contributed to the final approval of the article.

## Conflict of Interest

The authors declare that the research was conducted in the absence of any commercial or financial relationships that could be construed as a potential conflict of interest.
